# A novel function of *IMPA2*, plays a tumor-promoting role in cervical cancer

**DOI:** 10.1038/s41419-020-2507-z

**Published:** 2020-05-14

**Authors:** Kan Zhang, Lei Liu, Min Wang, Min Yang, Xianping Li, Xiaomeng Xia, Jingjing Tian, Shan Tan, Lingli Luo

**Affiliations:** 1 Department of Laboratory Medicine, Second Xiangya Hospital, Central South University, Changsha, Hunan China; 2 Department of Obstetrics and Gynecology, Second Xiangya Hospital, Central South University, Changsha, Hunan China

**Keywords:** Cervical cancer, Tumour biomarkers

## Abstract

Discovery of genes and molecular mechanism involved in cervical cancer development would promote the prevention and treatment. By comparing gene expression profiles of cervical carcinoma in situ (CCIS) and adjacent normal tissues, we identified a potential cancer-promoting gene, *IMPA2*. This study aimed to elucidate the role of *IMPA2* and underlying molecular mechanisms in cervical cancer progression. To do this expression of *IMPA2* was compared between human cervical cancer and corresponding adjacent normal cervical tissues firstly. CCK-8 assay, clone formation assay, wound healing assay, transwell assay, and tumor formation in nude mice were performed to demonstrate the effect of *IMPA2* in cervical cancer proliferation and metastasis. Further proteomic profiling and western blotting explored the molecular pathway involved in the *IMPA2*-regulating process. The results showed that *IMPA2* gene expression was upregulated in cervical cancer. Consistently, silencing of *IMPA2* suppressed tumor formation in BALB/c nude mice. Short hairpin RNA (shRNA)-mediated *IMPA2* silencing significantly inhibited proliferation and colony-forming abilities of cervical cancer cells, while *IMPA2* overexpression had little impact. Also, *IMPA2* silencing suppressed cellular migration, but overexpression promoted migration. Proteomics analysis revealed the involvement of mitogen-activated protein kinase (MAPK) pathway in tumor-promoting action of *IMPA2*. Significantly, the inhibition of *IMPA2* activated ERK phosphorylation, and its inhibitory effects can be restored by using selective ERK inhibitor, FR180204. In conclusion, *IMPA2* acts as an oncogene in the proliferation and migration of cervical cancer. *IMPA2* downregulated ERK phosphorylation to promote cervical cancer. These findings identify a new mechanism underlying cervical cancer and suggest a regulating effect of *IMPA2* in MAPK signaling pathway.

## Introduction

Cervical cancer is the fourth leading cause of cancer-associated death regarding gynecological malignancies worldwide^1^, even though prevention and treatment have rapidly developed recently^2,3^. As we all know, the most significant cause of cervical cancer is persistent infection of human papillomavirus (HPV). HPV is detected in 99% of cervical cancer patients^4^, but most women infected with HPV failed to develop invasive cervical cancer^5^. Therefore, comprehensive understanding of molecular mechanisms underlying cervical cancer will promote the earlier diagnosis and effective treatment.

Genetic mutations were proved to play important roles in development of cervical cancer^6^. RNA sequencing provides an efficient and comprehensive method to identify the key genes and molecular pathways involved in cervical cancer pathogenesis^7,8^. Cervical carcinoma in situ (CCIS), without stromal invasion, and superficially invasive carcinomatosis, is the earliest stage in cancer progression. By comparing gene expression profiles of CCIS and adjacent normal tissues, it is possible to look for the most direct evidence of tumorigenesis and help early diagnosis. Here, we sought potential oncogenes of cervical cancer, by comparing mRNA expression profiles of tissue samples of CCIS with adjacent normal cervical tissue. *IMPA2* gene was discovered to be significantly upregulated in CCIS tissues. *IMPA2* located on chromosome 18p11.2, encodes myo-inositol monophosphatase 2 (IMPA2) with 288 amino acids^9^. IMPA2 has intrinsic IMPase activity that is completely dependent on magnesium^10^, and is involved in phosphatidylinositol signaling pathway, which is associated with cellular activities such as metabolism, secretion, cell growth, and differentiation^11^. Therefore, we speculated that *IMPA2* may be a cancer-promoting gene in cervical cancer. However, most studies about *IMPA2* focused on neuropsychiatric diseases and the pharmacological action of Lithium^10,12,13^. Recently, French et al.^14^ found that *IMPA2* expression might affect accumulation of methotrexate polyglutamates (MTXPGs) in leukemia. In addition, Lin et al.^15^ indicated that *IMPA2* downregulation leads to poor outcomes in clear cell renal cell carcinoma (ccRCC). This is contrary to our speculation on the role of *IMPA2* in cervical cancers. As there are few published articles about the role of *IMPA2* gene in cervical cancer, this study performed both in vitro and in vivo studies to discuss the relationship between cervical cancer and *IMPA2*.

Here, we discussed the role of *IMPA2* and found that *IMPA2* promoted the ability of proliferation, metastasis, and in vivo tumorigenesis of cervical cancer cells. Further proteomic analysis was performed to discuss the possible mechanisms regulated by *IMPA2* in cervical cancer. The present study is proposed to identify the potential cancer-promoting action of *IMPA2* in cervical cancer and explore possible pathways controlled by *IMPA2* to further understanding the molecular mechanisms underlying cervical cancer.

## Material and methods

### Tissue sample selection

Cervical carcinoma in situ (CCIS) and adjacent normal tissues were obtained from three patients who underwent radical hysterectomy at the Second Xiangya Hospital, Central South University. All patients were diagnosed by multipoint biopsy. Table 1 showed the clinical characteristics of the patients. After tumor purity analysis performed by ESTIMATE algorithm, samples were collected for transcriptome analyses^16^. None of the three patients had received adjuvant therapy (chemotherapy or radiotherapy) prior to uterectomy. In addition, the other 58 patients with cervical cancer were enrolled from September 2015 to December 2017. Clinicopathologic features of the patients were shown in Table S1. Among them, 57.4% (35/61) were positive for HPV16, 19.7% (12/61) for HPV 58, 11.5% (7/61) for HPV 33, 11.5% (7/61) for HPV 52, 8.2% (5/61) for HPV51, and 8.2% (5/61) for HPV18. This study was approved by the Joint Ethics Committee of the Central South University Health Authority and performed following national guidelines. Written informed consent was obtained from all the patients. The clinical staging and clinicopathological classifications were determined according to the International Federation of Obstetrics and Gynecology (FIGO). Paired cervical cancer and adjacent normal tissues were collected at surgery, immediately frozen in liquid nitrogen and stored until total RNA or proteins were extracted.

**Table 1:** Clinical characteristic of cervical cancer patients enrolled in this study.

ID	Age at diagnosis (years)	Pathological type	FIGO stage	HPV types	Reproductive history (pregnancy-birth-abortion)
1	47	CIS	0	51+/52+/44+	5-1-4
2	51	CIS	0	33+/53+	5-2-3
3	43	CIS	0	16+/53+	2-1-1

*CIS* carcinoma in situ, *FIGO* International Federation of Gynecology and Obstetrics, *HPV* Human Papilloma Virus.

### Immunohistochemistry staining analysis

The immunohistochemical staining procedure was performed as previously described^17^. Cervical cancer samples for *IMPA2* detecting were obtained from the Second Xiangya Hospital of Central South University. Samples for ERK and p-ERK detecting were xenografts from mice. The staining positivity was determined by the following formula: IRS = intensityscore × quantity score. The percentage of positive cells was divided into five score ranks: <10% (0), 10–25% (1), 25–50% (2),50–75% (3), and >75% (4). The intensity of staining was divided into four score ranks: no staining (0), light brown(1), brown (2), and dark brown (3). Two different pathologists evaluated all the specimens in a blinded manner. The antibodies used were as follows: anti-IMPA2 rabbit monoclonal antibody (1:100, GeneCopoeis, USA); anti-ERK (1:500, Abcam, UK), and anti-pERK (1:400, Cell Signaling, USA).

### Cell culture

Cervical cancer cell line SiHa (#BNCC337881) and normal cervical epithelial cell line, HcerEpic (#BNCC340373) were purchased from the Cell Bank of BeNa culture collection (Beijing, China). Cervical cancer cell line HeLa (#GCC-UT0002CS) was purchased from the Cell Bank of Genechem (Shanghai, China). The cell line was cultured in Dulbecco’s modified eagle medium (DMEM) (Gibco, Grand Island, NY, USA) supplemented with 10% fetal bovine serum (FBS) (Gibco, Grand Island, NY, USA) and 1% antibiotics at 37 °C in an atmosphere containing 5% CO_2_.

### IMPA2 silencing

The short hairpin RNA (shRNA) targeting *IMPA2* mRNA (shIMPA2) and the negative control were obtained from Ribobio (Guangzhou, China). Sequences of shIMPA2 were listed as follow: forward 5′‘-CCGGGCCTTACAGACGATTAACTATCTCGAGATAGTTAATCGTCTGAAGGCTTTTTG-3′; Reverse 5′-AATTCAAAAAGCCTTACAGACGATTAACTATCTCGAGATAGTTAATCGTCTGTAAGGC-3′. The lentivirus was packaged using GV115 vector, pHelper 1.0 vector and pHelper 2.0 vector, as well as Lipofectamine 2000 (Invitrogen, Carlsbad, CA, USA; Thermo Fisher Scientific) for the HEK293T cell and then collected after 48 h. The SiHa cell line was infected with lentivirus and polybrene (1:500; Shanghai Ji Kai Gene Chemical Technology Co., Ltd., Shanghai, China) according to the manufacturer’s instructions. The expression change of *IMPA2* was determined by reverse-transcription polymerase chain reaction (RT-PCR) and Western blotting at 72 h after transfection.

### In vivo tumor formation assay

A total of 14 health female BALB/c (nu/nu) nudes (4 weeks, 20–23 g) were purchased from Shanghai Lingchang Biotechnology limited company and fed in SPF Animal Laboratory of Central South University with sterile water and food. The mice were divided into 2 groups based on the randomized table, Normal Control (shCtrl) group and *IMPA2* RNAi (shIMPA2) group. In all, 5 × 10^6^ indicated stable cell lines were subcutaneously injected into right flank of BALB/c (nu/nu) mice in each group. Tumor sizes and weights were measured once a week. Mice were examined by in vivo fluorescence imaging system (Lumina LT, Perkin Elmer, USA) and killed for the analysis of tumor burden after 4 weeks. The tumors were stripped for follow-up experiments. All the experiments were strictly accordant with the care and use guidelines of experimental animal and approved by the Animal Protection Committee.

### RNA isolation and quantitative real-time PCR

Total RNA was extracted using the Trizol reagent (Sangon Biotech, Shanghai, China). RNA (1 μg) was reverse transcribed into cDNA using Transcriptor First Strand cDNA Synthesis Kit (Roche Diagnostics, Germany) according to the supplier’s instructions. Quantitative real-time PCR analysis was performed with Stratagene Mx3000P qPCR system (Agilent Technologies, USA) using Thunderbird qPCR Mix (TOYOBO, Japan). CDNA samples were tested in triplicate and glyceraldehyde-3-phosphate dehydrogenase (GADPH) was used as a reference gene. The expression of *IMPA2* was quantified by measuring Ct values and normalized using the 2^−ΔΔCt^ method relative to GAPDH. The primer pairs used for qRT-PCR were designed using the primer3 program. Primers used were as follows: *IMPA2* forward, 5′-GAAACCTCTCTCGCAACTCAG-3′, reverse, 5′-GGGCAGGACAGATCATCAGAA-3′. GADPH forward: 5′- GAACGGGAAGCT CACTGG-3′, reverse, 5′-GCCTGCTTCACCACCT TCT-3′.

### Western blotting analysis

Details of Western blotting were previously described^18^. Cells at 80–90% confluence were lysed on ice in radioimmunoprecipitation assay buffer (RIPA; keygen biotech, China) containing PMFS complete protease inhibitor cocktail (keygen biotech, China). Protein concentration was determined by the BCA assay (keygen biotech, China). Equal protein samples (10 μg) were separated on 12% sodium dodecyl sulfate (SDS)/polyacrylamide gels, and transferred onto 0.45 µm polyvinylidene difluoride (PVDF) membranes (Immobilon-P; Millipore, Bedford, MA, USA). The antibodies used were as follow: anti-IMPA2 rabbit monoclonal antibody (1:1000; GeneCopoeis, USA); anti-ERK (1:10,000, Abcam, UK), anti-p38α (1:500, BBI China), anti-JNK1/2/3 (1:500, BBI China), p-ERK (1:500, Cell Signaling, USA), p-p38α (p-Thr180/Tyr182, 1:500, BBI China), and p-JNK1/2/3 (p-Th183/Ty185, 1:500, BBI China). Horseradish peroxidase (HRP)-conjugated goat anti‑rabbit immunoglobulin G (1:1000; BBI China) was used as second antibody and anti-GAPDH mouse monoclonal antibody (1:5000; BBI China) as a loading control. The final protein expression was detected by enhanced chemiluminescence (Bio-rad, Berkeley, CA, USA) according to the manufacturer’s suggested protocols. The band quantification was conducted using ImageJ (National Institutes of Health, Bethesda, MA, USA).

### CCK-8 cell viability assays

Cells were seeded into a 96-well plate at 2 × 10^3^ cells per well with 100 µl cultured medium and cultured for 24, 48, 72, and 96 h at 37 °C, 5% CO_2_. The cell viability was determined with CCK8 assay as previously described^19^. Each process was repeated three times.

### Colony formation assay

Cells (1000/well) were plated in 6-well plates and cultured for 2 weeks. The colonies were washed with PBS three times and fixed with 4% formaldehyde for 10 min. Then, the colonies were stained with 1% crystal violet for 10 min. After washing, the colonies were counted. The experiment was carried out in triplicate for each cell line.

### Wound healing and transwell migration assays

In the wound healing assay, cells (2×10^6^/well) were seeded in 6-well plates. When the cells were 90% confluent, they were serum-starved for 24 h. A linear wound was created in the confluent monolayer using a 10-μl pipette tip. The wounds were observed and photographed immediately (time 0) and thereafter at 48 (magnification, ×200). Details of transwell migration assay was described previously^20^, 2 × 10^4^ cells in 200 µl of serum-free medium were added to the top chamber of the transwell (8-μm pore size, BD Biosciences, New Jersey, USA). The bottom well contained growth medium with 20% FBS. After 24 h incubation at 37 °C, cells that had migrated to the lower face of the filters were fixed with 4% paraformaldehyde and stained with hematoxylin and finally counted under a magnification of ×200 (10 random fields/well). Each experiment was repeated at least 3 times.

### Sample preparation for proteomic measurement

The cultured cells were scraped and collected in 1.5 mL Eppendorf tubes. Then, samples were sonicated three times on ice using a high intensity ultrasonic processor (Scientz) in lysis buffer (8 M urea, 1% Protease Inhibitor Cocktail). The remaining debris was removed by centrifugation at 12,000×*g* at 4 °C for 10 min. Finally, the supernatant was collected and the protein concentration was determined with BCA kit. The protein solution was reduced with 5 mM dithiothreitol for 30 min at 56 °C and alkylated with 11 mM iodoacetamide for 15 min at room temperature in darkness. The protein sample was then diluted by adding 100 mM NH4HCO3 to urea concentration <2 M. Finally, trypsin was added at 1:50 trypsin-to-protein mass ratio for the first digestion overnight and 1 : 100 trypsin-to-protein mass ratio for a second 4-h-digestion.

### LC-MS/MS analysis

The tryptic peptides were separated by an EASY-nLC 1000 UPLC system and then subjected to NSI source followed by tandem mass spectrometry (MS/MS) in Q Exactive TM Plus (Thermo) coupled online to the UPLC. The resulting MS/MS data were processed using Maxquant search engine (v.1.5.2.8). Tandem mass spectra were searched against SwissProt Human database concatenated with reverse decoy database. Trypsin/P was specified as cleavage enzyme allowing up to two missing cleavages. The mass tolerance for precursor ions was set as 20 ppm in First search and 5 ppm in Main search, and the mass tolerance for fragment ions was set as 0.02 Da. Carbamidomethyl on Cys was specified as fixed modification, and oxidation on Met was specified as variable modifications. False positive rate (FDR) was adjusted to <1% and minimum score for peptides was set >40.

### Bioinformatics analysis of differentially expressed proteins

Firstly, protein ID of the differentially expressed proteins (DEPs) were converted to UniProt ID according to the UniProt-GOA database (www. http://www.ebi.ac.uk/GOA/) and then mapped to Gene Ontology (GO) IDs. Proteins were classified by GO annotation based on three categories: biological process, cellular component, and molecular function. Then, Kyoto Encyclopedia of Genes and Genomes (KEGG) database was used to annotate protein pathway and to identify enriched pathways. KEGG online service tools KAAS and KEGG mapper help annotate protein’s KEGG database description and map the annotation result on the KEGG pathway database. Finally, a two-tailed Fisher’s exact test were used to test the enrichment of the DEP against all identified proteins. A corrected *p*-value <0.05 was considered significant.

### Statistical analysis

The results were analyzed using SPSS 22.0 (Chicago, IL, USA) and GraphPad Prism 6 software (GraphPad Software, San Diego, CA, USA). The data were expressed as the mean ± SD. The *t*-test and one-way ANOVA Tukey’s post-hoc test were used to analyze the statistical significance of parametric data from two and more independent samples, respectively. Non-parametric Wilcoxon Signed-Ranks test, Friedman test, Mann–Whitney *U* test and Kruskal–Wallis test were used to analyze data from two related samples, three or more related samples, two independent samples and three or more independent samples, respectively.

## Results

### IMPA2 is overexpressed in cervical cancer

**Fig. 1:**
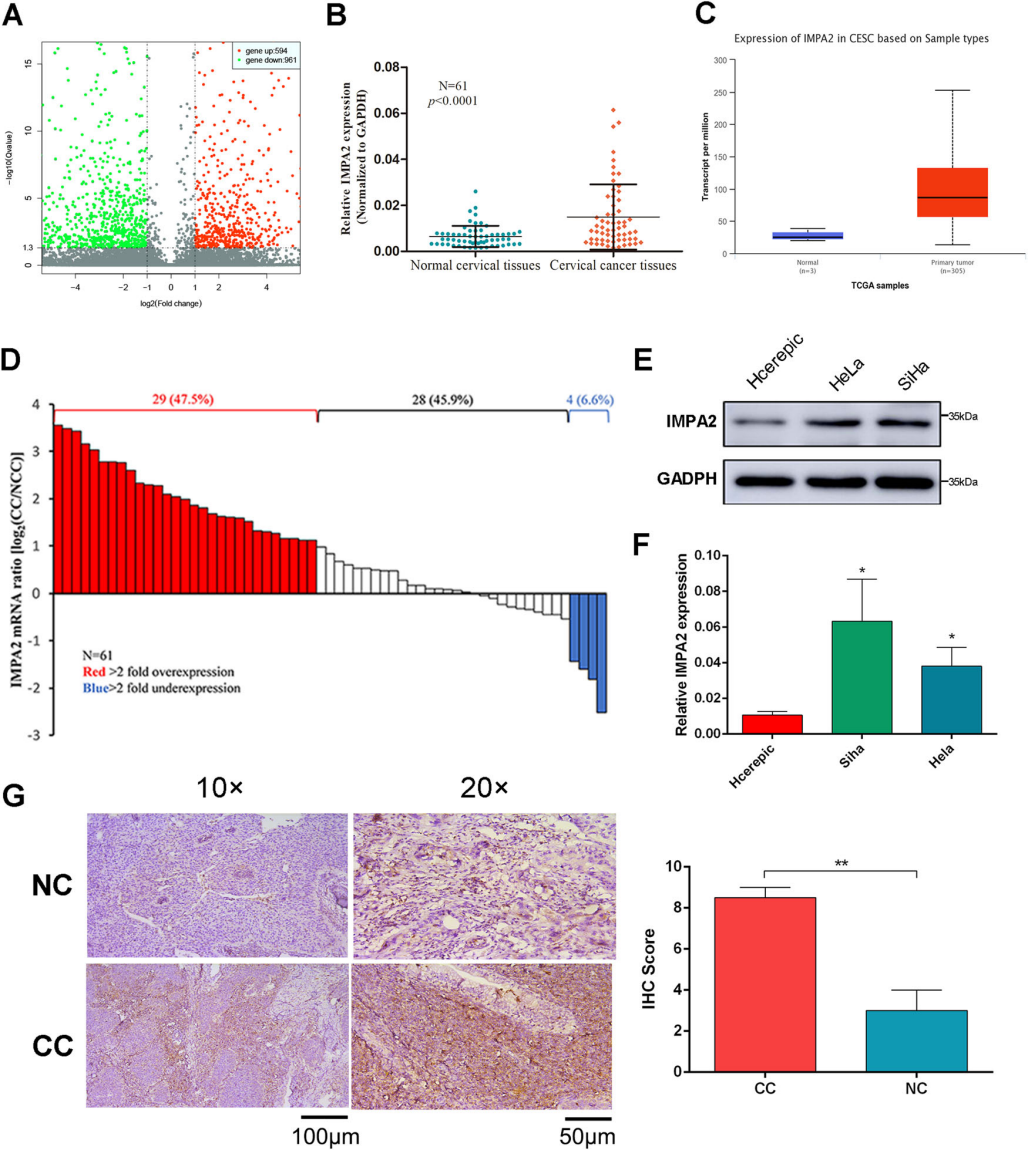
IMPA2 was overexpressed in cervical cancer tissue and cell lines. **a** Volcano plot of differentially expressed genes. **b** Relative expression of *IMPA2* in cervical cancer tissues (*n* = 61) compared with that of adjacent normal tissues (*n* = 61) (*P* < 0.0001). **c** Expression of *IMPA2* in normal and CESC tissues using public data from the TCGA-CESC dataset (Normal vs Primary tumor: *P* = 0.0004). **d** Waterfall plot analyses of *IMPA2* mRNA levels in CC and matched non-tumor cervical specimens. Red and blue bars represent samples that show a relative *IMPA2* fold change of ≥2 overexpression and underexpression, respectively (CC/NT). **e** *IMPA2* expression was determined using western blotting and qRT-PCR (**f**) in SiHa and Hela cells. **g** Immunohistochemical (IHC) detection of IMPA2 in normal cervical samples (NC) and cervical cancer samples and the Immunoreactivity scores of IMPA2 were shown. Scale bar, 100 or 50 μm. Values were all presented as mean values ± SD. *P < 0.05.

Comparing the gene expression profiles of CCIS and adjacent normal cervical samples resulted in 1555 differentially regulated genes at *P* < 0.05. Among the significant genes, 961 genes were up-regulated and 594 were down-regulated in CCIS (Fig. 1a). Of these, *IMPA2* gene was upregulated 11 times. Further real-time PCR was used to determine the *IMPA2* expression pattern in 61 pairs of cervical cancer tissues and their matched adjacent non-tumor cervical tissues. *IMPA2* is significantly overexpressed in cervical cancer samples (*P* < 0.0001, Fig. 1b). Overexpression of more than 2-fold was displayed in 47.5% (29 of 61) of the cervical cancer samples compared with non-tumor samples (Fig. 1d). Analysis of a public CESC (Cervical squamous cell carcinoma) dataset from The Cancer Genome Atlas (TCGA) (https://tcga-data.nci.nih.gov/tcga/tcgaDownload.jsp) also showed significant *IMPA2* overexpression (Fig. 1c)^21^. Expression of IMPA2 was also compared among cervical cancer cell lines and normal cervical epithelial cell line to prove the overexpression (Fig. 1e, f). Moreover, increased protein level of IMPA2 was also observed in paired cervical cancer samples by immunohistochemistry (IHC; Fig. 1g).

### Inhibition of IMPA2 suppresses tumorigenesis of SiHa cells in vivo

**Fig. 2:**
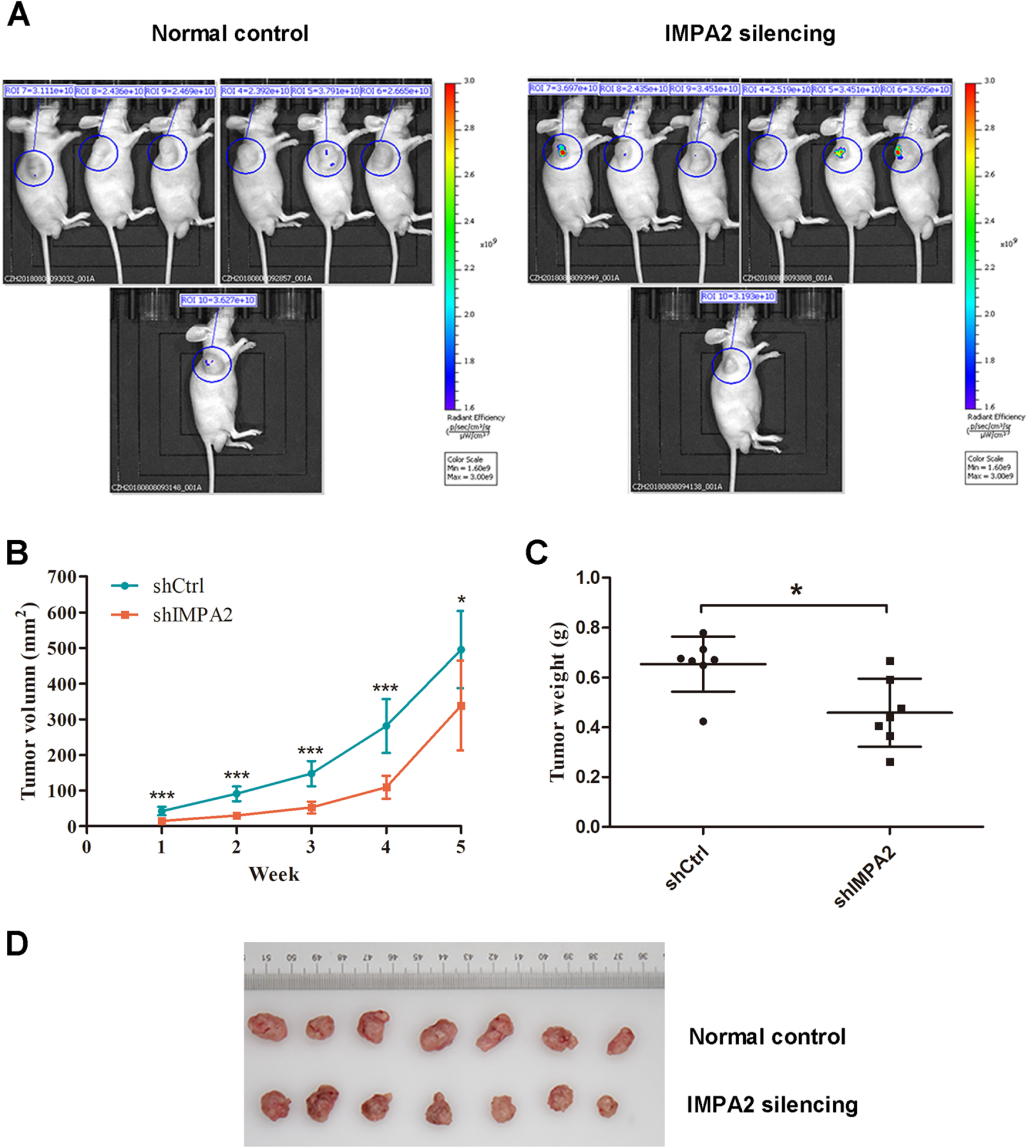
Inhibition of IMPA2 suppresses tumor growth in vivo. **a** Nude mice were transplanted subcutaneously with SiHa cells transfected with *IMPA2* shRNA or the control shRNA. After 35 days, the mice were killed. Whole-body fluorescent imaging was performed for each nude mouse, and the representative pictures was recorded and presented. **b** Tumor volumes were measured weekly from week one to five post-injection. **c** Tumor weights were measured after the mice were killed. Bars indicate mean values ± SD. **d** A representative picture of the morphology of tumor xenografts after excision at the 35th day. *P < 0.05.

SiHa cells infected with *IMPA2* shRNA or shRNA control were subcutaneously injected into each flank of nude mice. The tumor size of each nude mouse was measured and record weekly to draw the growth curve (Fig. 2b). After 5 weeks, all the mice were anesthetized to perform whole-body fluorescent imaging. As shown in Fig. 2a, no obvious metastasis was discovered in both groups. After that, all the mice were killed to harvest the xenografts. The tumor size and weight were measured and compared between groups. The *IMPA2* silencing group showed significantly smaller tumor size (*P* = 0.0274) and lighter average weight (*P* = 0.0123) than the control group (Fig. 2c, d).

### IMPA2 promotes the proliferation and clonogenicity of cervical cancer cells in vitro

**Fig. 3:**
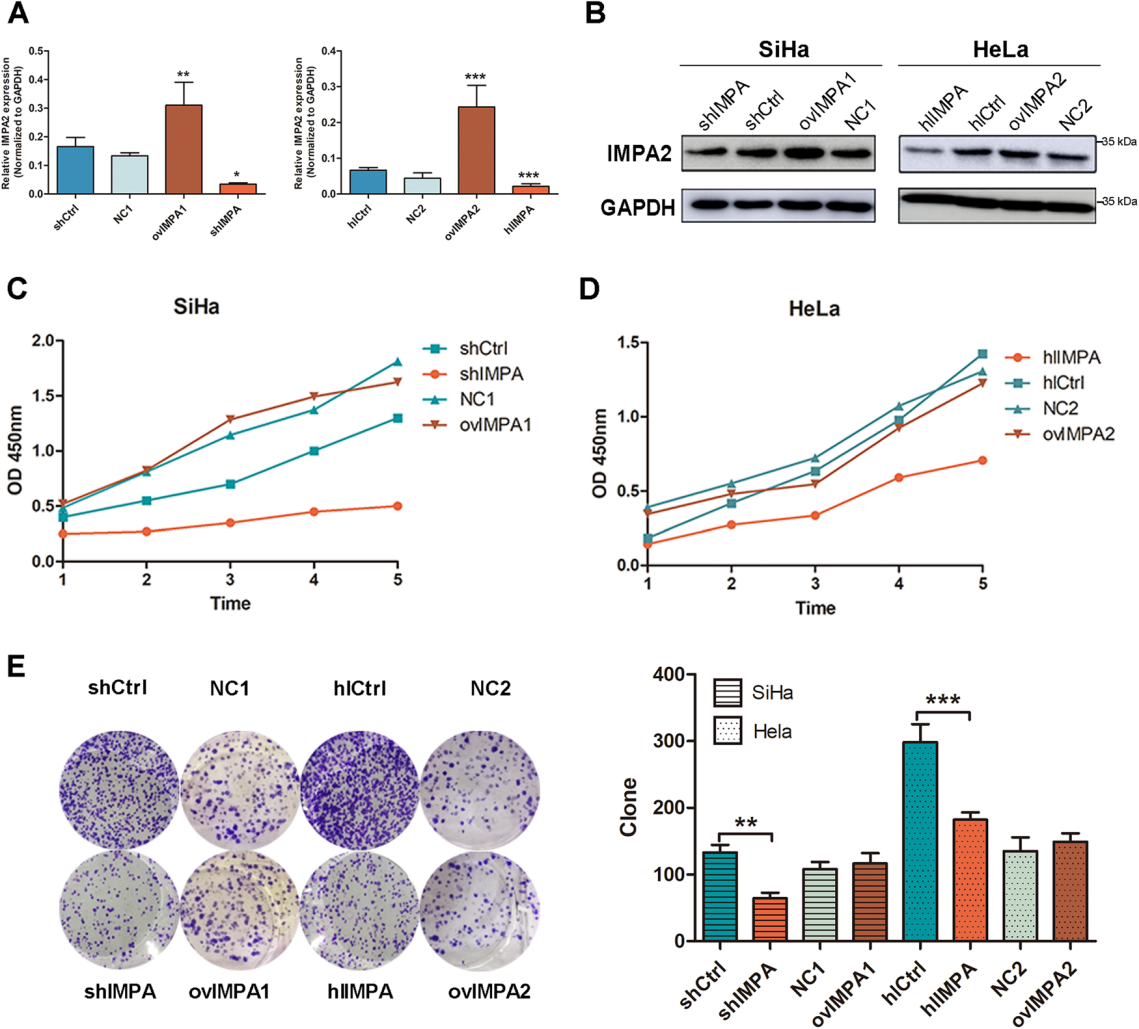
IMPA2 downregulation inhibits proliferation and clonogenicity of SiHa and Hela cells in vitro. **a**, **b** ShRNA silence of *IMPA2* dramatically downregulated *IMPA2* expression at the RNA level and protein level when compared to the negative control shRNA (shCtrl) in SiHa cell line by qRT-PCR and western blotting. **c**, **d** CCK-8 assay was performed to measure proliferation in *IMPA2* silencing and overexpressing SiHa or Hela cells and in the respective control cells. **e** The cell clonogenicity of *IMPA2* silencing and overexpressing SiHa or HeLa cells and the respective control cells was analyzed using Celigo Image Cytometry. The data represent the mean ± SDs of three replicates, ***P < 0.001.

The role of *IMPA2* in cervical cancer cells were detected after transfected with overexpressed plasmids and small interfering RNA (siRNA) lentivirus. The downregulation and overexpression efficiency were verified using qRT-PCR and western blotting (Fig. 3a, b). CCK8 cell viability assay (Fig. 3c, d) and colony formation assay (Fig. 3e) were performed to evaluate cell growth. As a result, silencing *IMPA2* in SiHa and HeLa cells both significantly slowed the cell growth. Overexpression of *IMPA2* failed to enhance the cell growth in both cervical cancer cells. Similarly, fewer and smaller colonies appeared in cells treated with *IMPA2*-siRNA than those in controls (*P* < 0.01). In contrast, no significant difference was found between the *IMPA2* overexpression group and the control group.

### IMPA2 promotes the migration of cervical cancer cells in vitro

**Fig. 4:**
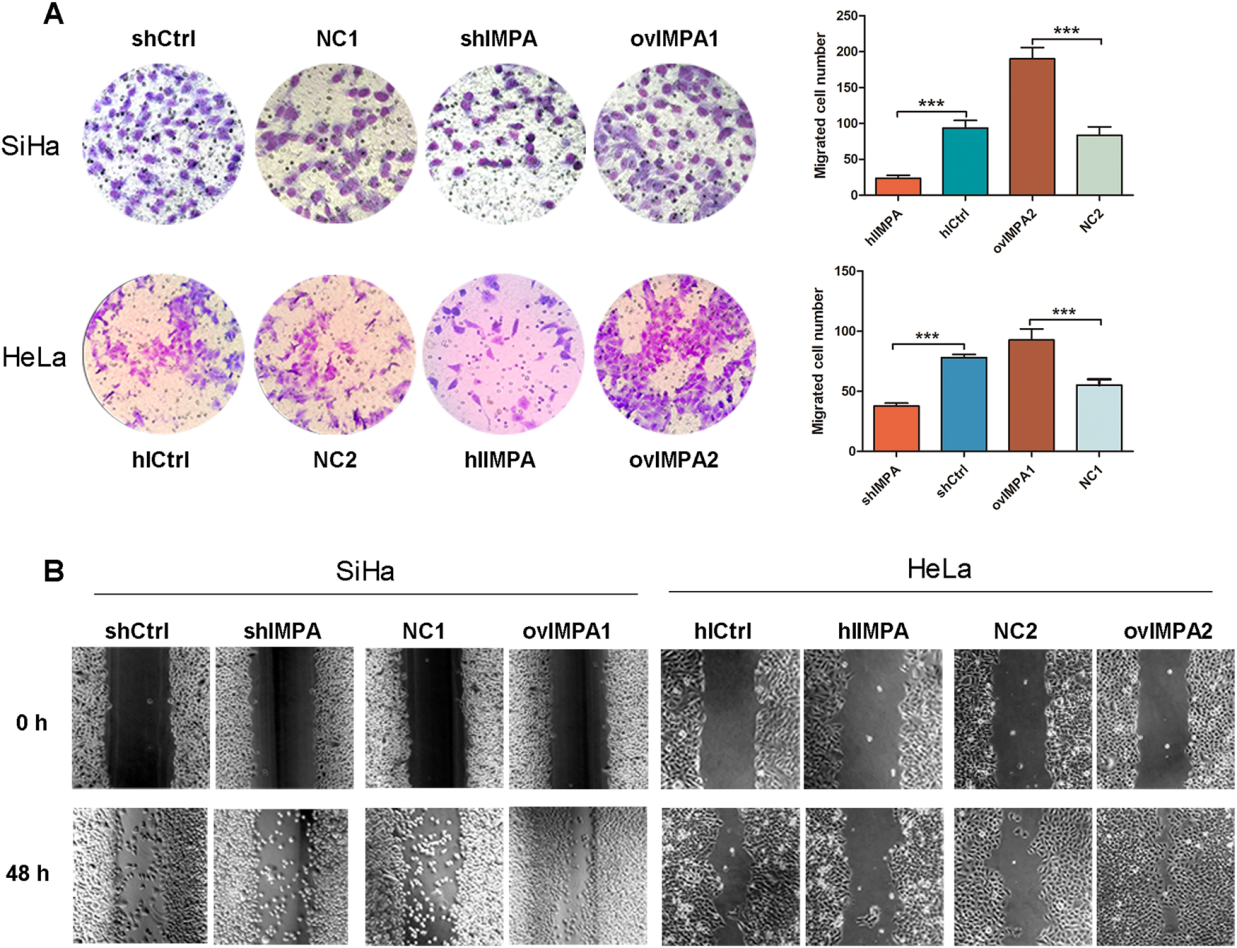
IMPA2 silencing blocks migration of SiHa and Hela cells in vitro. **a** Cell migration of *IMPA2* silencing and overexpressing SiHa or Hela cells and in the respective control cells was analyzed without Matrigel using transwell chamber. **b** Cell migration was analyzed by a wound healing assay. *IMPA2* silencing and overexpressing SiHa or Hela cells were seeded and grown to full confluence. In total, 48 h after creating the wound, the wounding space was observed and photographed. The data represent the mean ± SDs of three replicates. **P < 0.01; ***P < 0.001.

Wound-healing assay and transwell migration assay were conducted to examine the effect of *IMPA2* on the migration and invasion of cervical cancer cells. *IMPA2* silencing slowed the speed with which both cervical cancer cells filled the scratch, in comparison to the control in an obvious manner (Fig. 4b). The transwell assays yielded the similar results (*P* < 0.001, Fig. 4a). On the contrary, the cell migration capacity was improved (Fig. 4b) and the number of invaded cells were significantly higher (*P* < 0.001, Fig. 4a) in *IMPA2* overexpression group.

### Pathway analysis of differentially expressed protein between shCtrl and shIMPA2

**Fig. 5:**
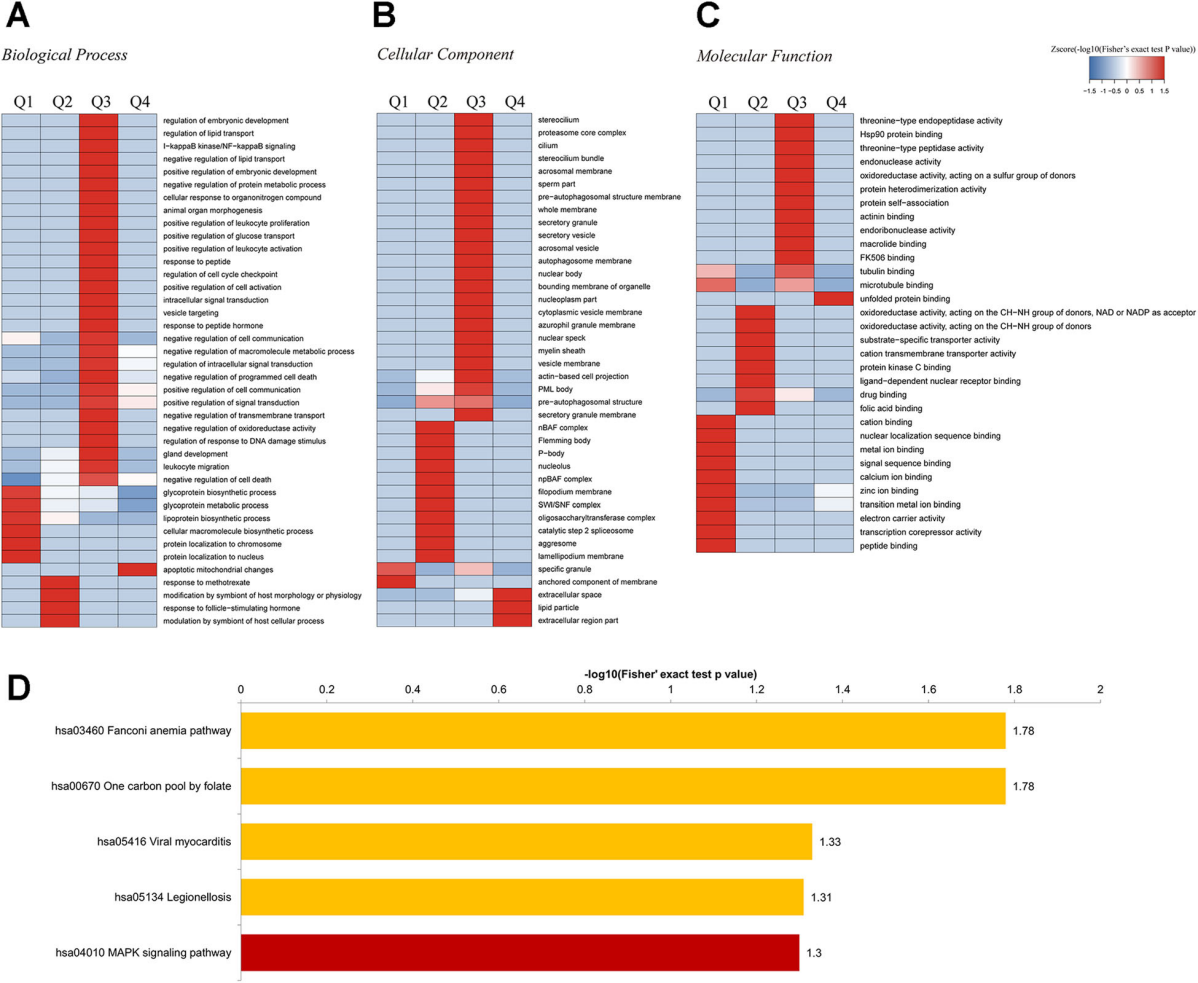
Proteomic profile after inhibition of IMPA2 in SiHa cells. **a**–**c** Functional enrichment of the differentially expressed proteins (DEPs) between shCtrl and shIMPA2 based on Gene ontology (GO) annotations. The results cover biologic processes (**a**), cellular component (**b**), and molecular function (**c**). **d** Enrichment of DEPs based on the KEGG pathway database. DEPs were divided into four parts according to the fold ratio of *IMPA2* expression level between shCtrl and shIMPA2. Q1, 0 < ratio ≤ 1/2; Q2, 1/2 < ratio ≤ 1/1.5; Q3, 1.5 < ratio ≤ 2; Q4, ratio > 2.

The proteomic profile of shCtrl and shIMPA2 (Fig. 5) was determined by LC-MS/MS. A total of 4364 proteins were identified. Using a cutoff value 1% FDR for statistical significance, 267 proteins were found to be differentially expressed: 112 significantly overexpressed (shIMPA2/shCtrl ratio ≥1.50) and 155 underexpressed (shIMPA2/shCtrl ratio ≤0.50). Further bioinformatics analysis was then performed to assess whether the DEPs were related to specific molecular pathways. In the Gene Ontology (GO) enrichment, 14 biological processes, 8 molecular functions, and 8 cellular components were enriched with statistical significance (Fig. 5a–c). Main biological processes include DNA repair, glycoprotein biosynthesis and metabolism, RNA biosynthesis, and cell death process. The analysis by cellular components showed that *IMPA2* silencing produced a more profound impact on autophagosome-related structure and secretory granule membrane. Moreover, the analysis of molecular functions showed that proteins involved in the binding of transcript, protein kinase and drug, and folic acid, as well as many enzymatic activities like oxidoreductase and DNA polymerase. Cluster analysis based on shIMPA2/shCtrl ratio showed the involved biological processed in more detail (Fig. 5b). KEGG pathway enrichment analysis indicated that most of the DEPs were related to Fanconi anemia pathway, one carbon pool by folate, viral myocarditis, legionellosis, and MAPK signaling pathway (Fig. 5d). Eight DEPs involved in the MAPK pathway were significantly changed after inhibition of *IMPA2*, including four upregulated proteins (HSPA1L, RAP1A, NFKB1 and TAB1) and four downregulated proteins (MAP2K3, EPHA2, IL1RAP, and ECSIT). The fold change and primary information of these proteins were detailed in Table 2.

**Fig. 6:**
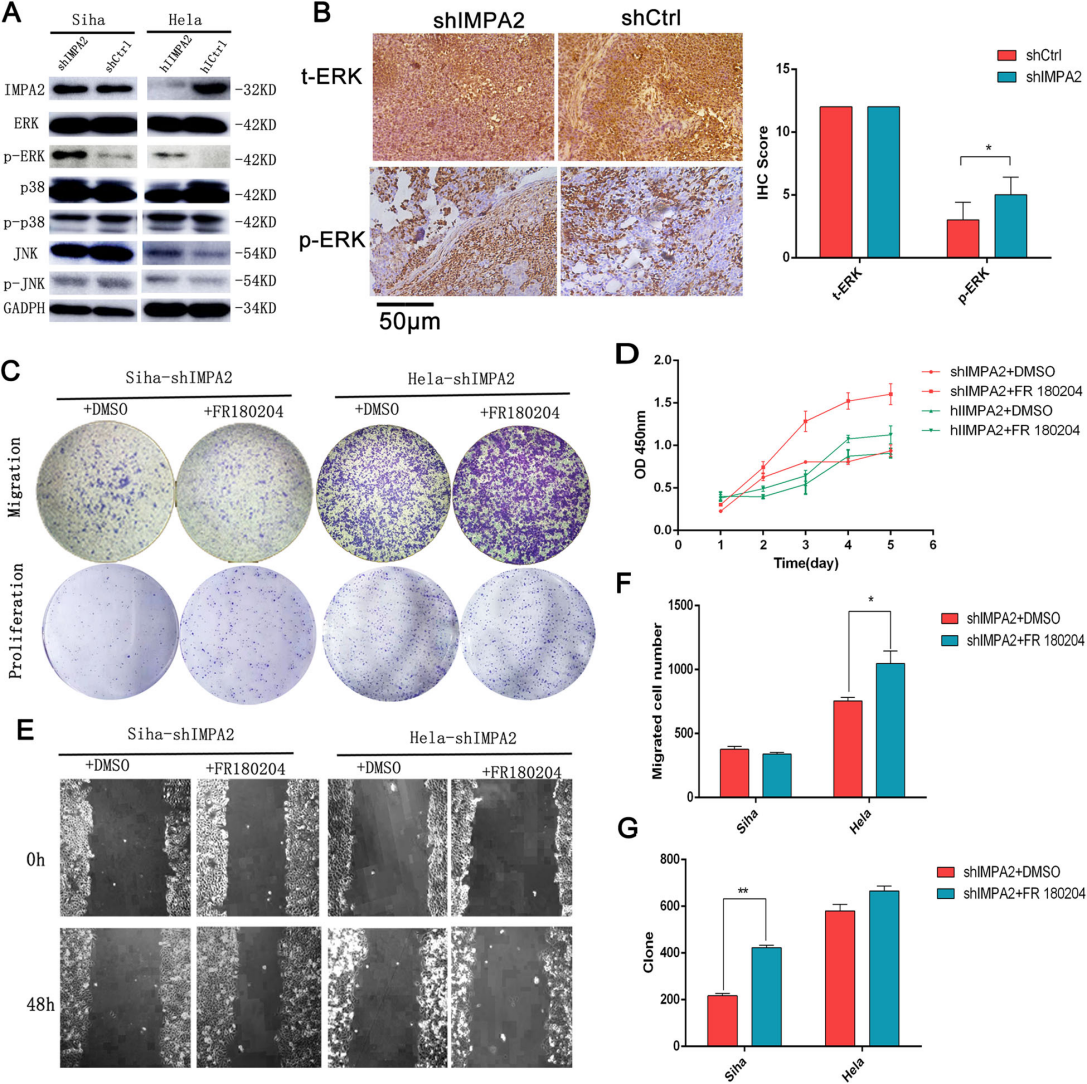
Silenced IMPA2 activated ERK and the IMPA2-induced cell proliferation and migration were revised by ERK inhibitor. **a** The expression levels of IMPA2 and MAPK signaling molecules ERK, p-ERK, p38, p-p38, JNK, p-JNK, and GAPDH protein in *IMPA2* silencing SiHa or HeLa cells were detected by western blotting. **b** ERK and p-ERK expression of the inxenografts were detected by immunohistochemical staining and the immunoreactivity scores of t-ERK and p-ERK were shown. Scale bar, 50 μm. The migratory and proliferation potential of *IMPA2* silencing SiHa or HeLa cells treated with FR 180204 or DMSO were analyzed by the transwell cell migration and colony formation assay (**c**). CCK-8 assay (**d**), wound healing assay (**e**). Number of migratory cells (**f**) and clone (**g**) was shown as mean ± SD from three independent experiments using triplicate measurements and statistically analyzed with Student’s *t*-test in each experiment. *p < 0.05, **p < 0.01.

**Table 2:** Primary information and fold change of 8 DEPs involved in the MAPK pathway.

Gene name	Protein accession	Protein description	SI/SN ratio
HSPA1L	P34931	Heat shock 70-kDa protein 1-like	2.016
TAB1	Q15750	TGF-beta-activated kinase 1 and MAP3K7-binding protein 1	1.823
RAP1A	P62834	Ras-related protein Rap-1A	1.573
NFKB1	P19838	Nuclear factor NF-kappa-B p105 subunit	1.551
EPHA2	P29317	Ephrin type-A receptor 2	0.644
MAP2K3	P46734	Dual specificity mitogen-activated protein kinase kinase 3	0.64
IL1RAP	Q9NPH3	Interleukin-1 receptor accessory protein	0.587
ECSIT	Q9BQ95	“Evolutionarily conserved signaling intermediate in Toll pathway, mitochondrial”	0.511

*SI* scilenced IMPA2 group, *SN* negative control group.

### Inhibition of IMPA2 activates the ERK/MAPK pathway

To understand further the mechanisms responsible for *IMPA2*-related cancer progression, MAPK signaling pathways were analyzed by Western blotting based on the proteomic profile results. We found that compared with the shCtrl group, *IMPA2* silencing significantly increased ERK phosphorylation in SiHa and Hela cells. Total levels ERK showed no statistical differences. And *IMPA2* had little effect on JNK and p38 phosphorylation (Fig. 6a). Furthermore, silenced *IMPA2* activating ERK expression was also been verified in inxenografts by Immunohistochemical staining (Fig. 6b). Thereafter, ERK inhibitor FR180204(10 μmol) was added in *IMPA2*-silenced SiHa and Hela cells for 48 h. We found that FR180204 treatment significantly reversed the *IMPA2*-induced decrease in cell viability (Fig. 6d), cell migration and invasion in Hela cell (Fig. 6c, e), suggesting that activation of the ERK signaling pathway may result in inhibitory of proliferation and migration induced by *IMPA2* in cervical cancer.

## Discussion

Cervical cancer progression is a continuous process from normal cervical to cervical intraepithelial neoplasia (CIN) and finally to cancer. Various molecules were involved^22–25^ besides the key role of human papillomavirus (HPV) infection. *IMPA2* gene is a protein-coding gene for myo-inositol monophosphatase 2^9^. This monophosphatase is one of the key enzymes in inositol phosphate metabolism and finally convert monophosphate into free inositol through dephosphorylation. *IMPA2* is received in many studies to be associated with neuropsychiatric diseases, like schizophrenia^26^, epilepsy^27^, bipolar disorder^11,28,29^, and Huntington’s disease^12,30^. Also, IMPA2 inhibition is accepted as the therapeutic mechanism of lithium in bipolar disorders^31^. However, there is only one published article clearly demonstrated the relationship between *IMPA2* expression and cancer progression. Lin et al. recently showed that *IMPA2* downregulation constitutes a novel signature for cancer metastasis and poor outcomes in ccRCC^15^. On the contrary, our data of RNA sequencing (RNA-seq) showed an obvious upregulation of *IMPA2* in cervical cancer. Therefore, our study investigated its ability to act as an oncogene and the possibility to be a potential diagnostic and prognostic biomarker as well as a therapeutic target in cervical cancer. In the present study, *IMPA2* was upregulated in cervical cancer tissues when compared with pair matched normal tissues, which is consistent with the dataset of *IMPA2* from TCGA. And about 47.5% (29/61) overexpressed more than 2-fold. However, the upregulation of *IMPA2* failed to correlate with clinical characteristics, HPV types and overall survival.

Our results demonstrated that inhibition of *IMPA2* could slower the proliferation and promote the migration of SiHa and Hela cells (Figs. 3, 4). And this effect of promoting tumorigenesis was also confirmed in vivo (Fig. 2). However, the in vivo result showed no migration or invasion in our shCtrl or shIMPA2 group. This can partly explain why *IMPA2* was not associated with clinical and pathological characteristics well (Table S1). And as introduced previously, *IMPA2* was identified upregulated in carcinoma in situ of cervix. We suspect that *IMPA2* can be a promoting element in the early stage of cervical cancer, but the further migration or invasion still need other contributing factors. Collectively, the present study is the first report to demonstrate that *IMPA2* may act as an oncogene in cervical cancer.

As the mechanism of promoting effect of *IMPA2* has not been studied yet, we first traced back to the researches about mechanisms behind the role of *IMPA2* in neuropsychiatric diseases. Most of the studies agreed that inhibition of *IMPA2* might induce IP3 accumulation and /or inositol depletion, which can finally regulate autophagy to help treat neurodegenerative diseases or bipolar disorders^12,30,31^. However, the mechanism of *IMPA2* in cancer is utterly unknown. We therefore performed proteomics analysis to look for clues of molecular regulation by *IMPA2* in cervical cancer. In all, 267 proteins with different expression were detected, 149 of which have previously reported to be potentially related to biological processes of DNA damage repair, cell death, glycoprotein biosynthesis and metabolism, RNA biosynthesis, and embryonic development. Clustering analysis showed the involvement of cell death in more detail, as regulation of cell death, programmed cell death, and apoptotic mitochondrial changes were all significantly clustered.

The KEGG enrichment analysis for the identification of pathways unveils possible involvement of MAPK signaling pathway. MAPK plays an important role in regulating cell proliferation^32^, differentiation^33,34^, cell cycle arrest^35^, apoptosis^24,36,37^, immune function^38^, and autophagy^36,39^. The MAPK signaling pathway is shared by four distinct cascades^40^, including Jun amino-terminal kinases (JNK1/2/3), extracellular signal-related kinases (ERK1/2), p38-MAPK and ERK5. The expression of above key molecules of SiHa and Hela cells pre and post inhibition of *IMPA2* were compared by western blotting. Phosphorylation of ERK was activated after *IMPA2* silencing. JNK 1/2/3 and p38 remained unchanged (Fig. 6), suggesting that MAPK/ERK signaling pathway take part in *IMPA2*-induced tumorigenesis of cervical cancer.

**Fig. 7:**
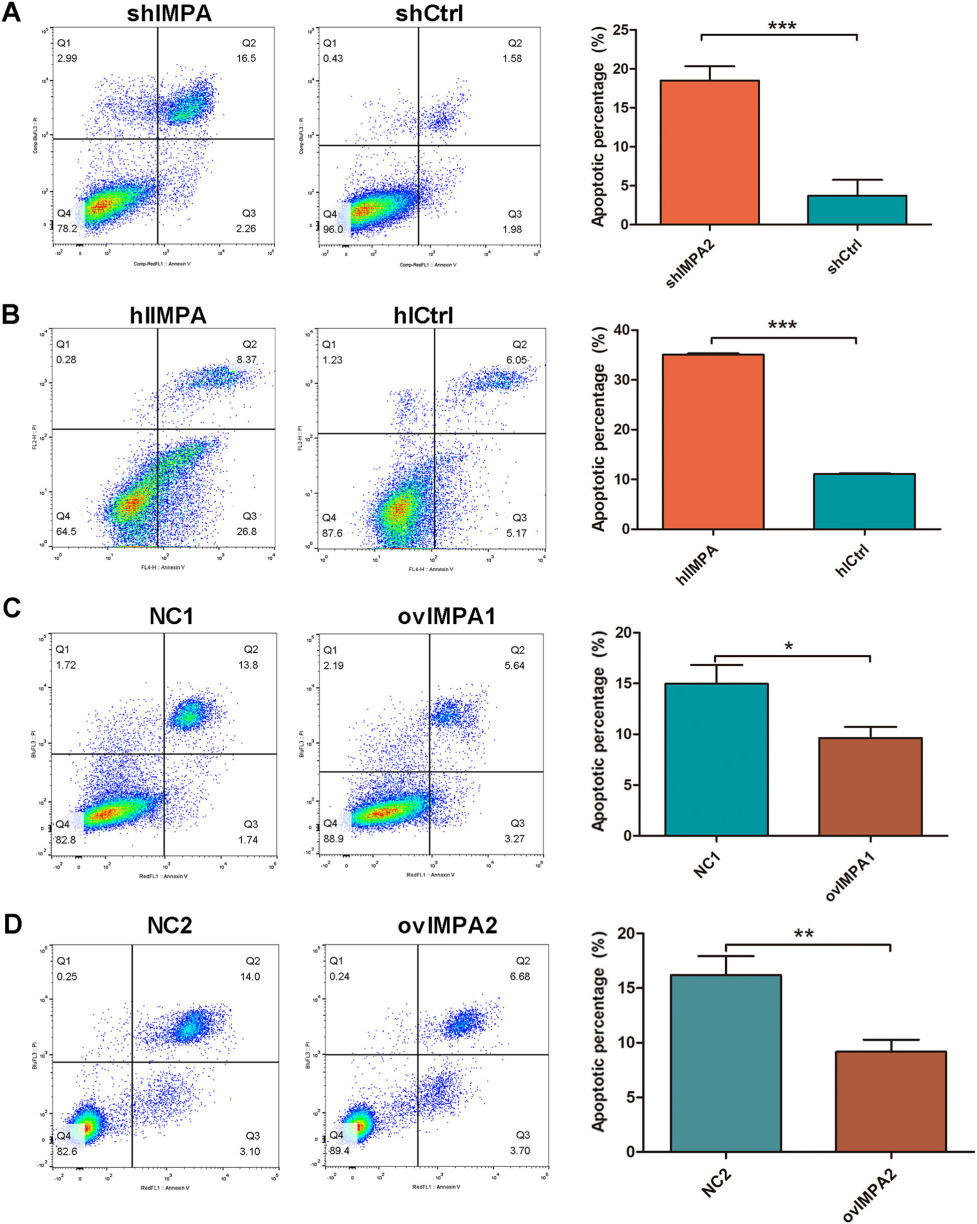
IMPA2 silencing induced apoptosis of SiHa and Hela cells. **a** Flow cytometry analysis of apoptosis showed that the knockdown of *IMPA2* in SiHa cell elevated the percentage of apoptotic cells, and which was decreased when *IMPA2* was overexpressed (**b**). The consistent results have been observed in *IMPA2* silencing or overexpressing Hela cells (**c**, **d**).

Many researches about chemotherapeutic drugs^41,42^, tumor-related gene^43^, microRNA^38^, and LncRNA^44^ have shown multiple effects on cervical cancer by regulation of MAPK signaling pathway. However, most of the studies agreed that phosphorylation of ERK^38,45^ was upregulated to promote the proliferation or (and) suppress the apoptosis^33,46^ of cervical cancer, while evidences showed contrary effect in this study (Fig. 6). When added ERK inhibitor in *IMPA2*-silenced cells, we could find that the proliferation capacity of Hela and migration of SiHa could not be revised completely (Fig. 6f, g). Therefore, we thought ERK cannot regulate tumorigenesis directly. Previous studies demonstrated that activation of ERK MAPK could also induce cytotoxicity or tumor inhibition in some cancers^19,41,47^. Some studies revealed that activation of ERK could promote apoptosis progress^48,49^. And as we all know, apoptosis is a common process of programmed cell death and plays a key role in maintaining cellular homeostasis in normal and cancer cells^35,50^. Therefore, we suspect that upregulated ERK decreased cervical cancer progression by activating apoptosis in *IMPA2* silencing cells. And the primary apoptosis assay by using flow cytometry had initially verified our hypothesis (Fig. 7), but this hypothesis needs to be confirmed in a further study.

In conclusion, *IMPA2* is a novel tumor promotor that regulates ERK MAPK in cervical cancer. Thus, silencing *IMPA2* could step down proliferation and metastasis of cervical cancer cells. It could also induce activation of ERK. These findings provided promising insights into developing novel cancer therapies by inhibiting the *IMPA2* in cervical cancer.

## Supplementary information

Correlation of the expression of IMPA2 with clinicopathologic features.

Results of tumor sample purity assessment

table of differentially expressed protein statistics of proteomics
